# Altered resting-state intra- and inter- network functional connectivity in patients with persistent somatoform pain disorder

**DOI:** 10.1371/journal.pone.0176494

**Published:** 2017-04-28

**Authors:** Zhiyong Zhao, Tianming Huang, Chaozheng Tang, Kaiji Ni, Xiandi Pan, Chao Yan, Xiaoduo Fan, Dongrong Xu, Yanli Luo

**Affiliations:** 1 MOE & Shanghai Key Laboratory of Brain Functional Genomics (East China Normal University), Institute of Cognitive Neuroscience, Shanghai Key Laboratory of Magnetic Resonance Institute of Cognitive Neuroscience, East China Normal University, Shanghai, China; 2 Department of General Psychiatry, Shanghai Changning Mental Health Center, Shanghai, China; 3 Department of Rehabilitation Medicine, Huashan Hospital, Fudan University, Shanghai, China; 4 Department of Psychiatry, HongKou District Mental Health Center of Shanghai, Shanghai, China; 5 Department of Psychiatry, Tongji Hospital of Tongji University, Shanghai, China; 6 Shanghai Key Laboratory of Brain Functional Genomics (MOE & STCSM), School of Psychology and Cognitive Science, East China Normal University, Shanghai, China; 7 Department of Psychiatry, University of Massachusetts Medical School, Worcester, Massachusetts, United States of America; 8 MRI Unit, New York State Psychiatric Institute and Columbia University, New York, NY, United States of America; 9 Department of Psychological Medicine, Renji Hospital, School of Medicine, Shanghai Jiaotong University, Shanghai, China; Brainnetome Center & The National Laboratory of Pattern Recognition, CHINA

## Abstract

Patients with persistent somatoform pain disorder (PSPD) usually experience various functional impairments in pain, emotion, and cognition, which cannot be fully explained by a physiological process or a physical disorder. However, it is still not clear for the mechanism underlying the pathogenesis of PSPD. The present study aimed to explore the intra- and inter-network functional connectivity (FC) differences between PSPD patients and healthy controls (HCs). Functional magnetic resonance imaging (fMRI) was performed in 13 PSPD patients and 23 age- and gender-matched HCs. We used independent component analysis on resting-state fMRI data to calculate intra- and inter-network FCs, and we used the two-sample t-test to detect the FC differences between groups. Spearman correlation analysis was employed to evaluate the correlations between FCs and clinical assessments. As compared to HCs, PSPD patients showed decreased coactivations in the right superior temporal gyrus within the anterior default-mode network and the anterior cingulate cortex within the salience network, and increased coactivations in the bilateral supplementary motor areas within the sensorimotor network and both the left posterior cingulate cortex and the medial prefrontal cortex within the anterior default-mode network. In addition, we found that the PSPD patients showed decreased FNCs between sensorimotor network and audio network as well as visual network, between default-mode network and executive control network as well as audio network and between salience network and executive control network as well as right frontoparietal network, and increased FNCs between sensorimotor network and left frontoparietal network, salience network as well as cerebellum network, which were negatively correlated with the clinical assessments in PSPD patients. Our findings suggest that PSPD patients experience large-scale reorganization at the level of the functional networks, which suggests a possible mechanism underlying the pathogenesis of PSPD.

## Introduction

Patients with persistent somatoform pain disorder (PSPD) often suffer from persistent, severe and distressing pain without fully explanatory peripheral pathology. In the recent ICD-11 beta draft, PSPD is subsumed to bodily distress disorder (BDD), a disorder characterized by bodily symptoms that are distressing to the individual and attention directed toward those symptoms that far exceeds what is warranted in relation to the symptoms’ nature and progression [1]. Numerous studies have demonstrated that the pain system of human brain is involved in the widely distributed regions, including the somatosensory cortices, thalamic nuclei, anterior cingulate cortex (ACC), prefrontal cortex (PFC), insular cortex, and the default mode network (DMN) (medial prefrontal cortex (mPFC), posterior cingulate cortex (PCC) and posterior parietal cortex), which are associated with the sensory-motivational and affective-discriminative aspects of pain [2–7]. So far, most of the studies related to PSPD focus on the behavioral changes in PSPD patients. By utilizing the Toronto Alexithymia Scale and the Hospital Anxiety and Depression Scale, the study [8] found that adolescents with PSPD had higher levels of alexithymia and anxiety than healthy ones had. Another study [9] used a series of animated morph clips to find that although PSPD patients’ ability to recognize facial expressions was normal, they exhibited deficits in mind-reading abilities. Additionally, a recent study [10] showed that PSPD patients scored lower relative to the general population on the SF-36 scale measuring quality of life, which correlated with the pain, depression and anxiety scores. However, these studies do not uncover the brain mechanism underlying the pathogenesis of PSPD.

Resting-state functional magnetic resonance imaging (fMRI) is a task-independent and noninvasive method to assess brain regional and neural circuitry function. The low-frequency (0.01–0.08 Hz) fluctuations of the blood oxygen level dependent (BOLD) signal detected during resting state by fMRI are considered to reflect spontaneous neuronal activities [11–14]. Recently, we used the regional homogeneity method on the resting-state fMRI data to find that, compared with healthy controls (HCs), the PSPD patients showed increased ReHo in the PFC and inferior parietal lobe (IPL), and they showed decreased ReHo in the somatosensory cortex as well as posterior cerebellum. The mean ReHo of IPL in the PSPD patients correlated with the clinical assessments [15]. In order to further improve our understanding for the brain mechanism of PSPD, there will need to be more studies investigating neuro-mechanisms. It is well-known that the traditionally resting-state functional connectivity (FC) is primarily applied to explore interregional temporal correlations of low frequency fluctuations in BOLD signals [16]. Generally, in the analysis, we first choose a region of interest (ROI) as the seed. Second, we calculate the reference time course by averaging the time series of all voxels in the seed ROI. Finally, we perform a Pearson’s correlation analysis, namely FC, between the reference time course and time series of each voxel in the brain. Therefore, the traditional method only detects the FC between two regions, but it fails to explain the change in the intra- and inter-network connectivity of the whole brain. The present study explores the large-scale functional organization of the brain in PSPD patients by investigating the intra- and inter-network connectivity.

Independent components analysis (ICA) have revealed aberrant resting-state networks (RSNs) in many disorder studies, such as schizophrenia [17], congenital blindness [18], amyotrophic lateral sclerosis [19] and somatoform pain disorder [20]. Nevertheless, the alterations in the FC within and across RSNs in PSPD patients are still unknown. From the perspective of pain perception, we hypothesize that the intra-network FCs in PSPD patients are altered in those networks associated with sensation, emotion and cognition. Because the changed FCs have been reported between the brain regions of pain systems and those belonging to other RSNs in some chronic pain disorders [3,4,20], we also hypothesize that the pain-related inter-network FCs are altered in PSPD patients. In the present study, we used an ICA-based RSN analysis to investigate whether both intra- and inter-network FCs are altered in PSPD patients.

## Materials and methods

### Participants

Thirteen patients were consecutively recruited from the Department of Psychosomatic Clinic of Tongji University between May 2012 and May 2015. Two trained psychiatrists (Yanli Luo and Tianming Huang) diagnosed the PSPD using Mini International Neuropsychiatric Interview (M.I.N.I) to exclude other psychiatric conditions and severe somatic comorbidities, such as diabetes, hypertension, tumor, and hyperlipidemia. Inclusion criteria for PSPD patients included: 1) right-hand dominance (The hand dominance was tested by using the Edinburgh Handness Inventory [21]); 2) ages between 18 and 65 years; 3) duration of clinical pain of at least 6 months; 4) diagnosis of PSPD according to ICD-10 criteria. Exclusion criteria for all subjects included current or past history of any of the following as indicated: 1) presence of pain symptoms due to severe somatic diseases; 2) existence of uncontrolled diseases, such as congestive heart failure, hypertension, cerebrovascular disease, thyropathy; 3) substance or depilatory abuse, such as alcohol and cocaine; 4) presence of mental diseases (such as affective disorder, suicidal depression, anxiety disorder, phobic anxiety disorder, obsessive compulsive disorder, and posttraumatic stress disorder); 5) electroconvulsive therapy within past 4 weeks; 6) current pregnancy; 7) participation in other clinical trials within past 4 weeks; 8) indications as evidenced by magnetic resonance imaging (MRI) of cerebral atrophy diagnosed by a radiologist who visually analyzed the MRI images on the scene. In most cases, participants’ self-reported pain was diffuse. The predominant clinical pain in the patient group was located in the 1) head, neck, and face region (diffuse pain, n = 4); 2) lower back region (lower back pain, n = 1); 3) pelvic region (lower abdomen pain, n = 4); and 4) upper and lower limbs (n = 2). Two patients reported more than one predominant pain location (one patient with lower abdomen pain as well as neck pain, and one patient with pain in the lower limbs along with back pain). There was no significant difference (t = -0.10, p = 0.92) in the VAS score before and after scanning in 13 patients. We considered the patients as severely affected for the diagnostic group of PSPD from a clinical point of view. All patients were previously exposed to Duloxetine with a maximum dose of 60mg/day or Venlafaxine with a maximum dose of 150mg/day, and they underwent MRI scanning after a washout period of 7 days based on the half-life of most antidepressants being used in previous studies [22,23]. Additionally, 23 HCs matched in age, gender, and handedness were recruited (Table 1). All HCs had no chronic/ongoing pain and did not meet ICD-10 criteria for any psychiatric disease. Demographic characteristics and clinical assessment of individual for PSPD patients and HCs was displayed in the S1 Table.

**Table 1:** Demographic characteristics and clinical assessment of participants.

	HCs (n = 23)	Patients (n = 13)	Patients vs HCs
	mean ± std	mean ± std	p-value
Age (years) ^a^	46.1 ± 12.7	46.0 ± 14.3	0.99
Sex(male: female) ^b^	12:11	5:8	0.84
Hand dominance	R	R	-
Duration of illness (years) ^a^	-	3.5 ± 2.57	-
VAS^a^	-	5.69 ± 2.10	-
SAS^a^	-	35.62 ± 9.42	-
SDS^a^	-	37.77 ± 8.57	-
FD^a^	0.08 ± 0.04	0.06 ± 0.03	0.25

a: Independent t-test

b: Chi-Square test.

VAS: Visual Analogue Scale; SAS: Self-Rating Anxiety Scale; SDS: Self-Rating Depression Scale; FD Frame-wise Displacement.

This study was approved by the local Ethics Committee of Tongji Hospital of Tongji University and conducted in accordance with the Declaration of Helsinki. Written informed consent was obtained for all the participants.

### Clinical assessments

The Visual Analogue Scale (VAS), Zung Self-Rating Anxiety Scale (SAS), and the Zung Self-Rating Depression Scale (SDS) were used to assess pain characteristics, anxiety and depressive symptom, respectively, in each patient. Detailed descriptions of these three assessments were elaborated in our previous study [15].

### MRI data acquisition

The MR images were acquired with a Siemens Trio 3.0 Tesla MRI scanner (Siemens, Erlangen, Germany) at the Shanghai Key Laboratory of Magnetic Resonance, East China Normal University. All patients were asked to discontinue their pain medications 1–3 days before fMRI. Before fMRI, the participant’s head was stabilized using foam pads to minimize both head movement and scanner noise. Resting-state fMRI of the whole brain was performed using an echo-planar imaging (EPI) sequence: axial slice: 33, slice thickness: 4 mm, no gap, matrix: 64 × 64, repetition time: 2,000 ms, echo time: 30 ms, flip angle = 90°, and field of view: 192 mm × 192 mm. During the fMRI scanning, all subjects were instructed to keep their eyes closed, relax, and movements as little as possible without thinking about anything in particular. Each scan lasted for 8 minutes and 6 seconds, but the first six seconds would comprise of dummy scanning. Thus, a total of 240 image volumes were collected. Additionally, T1-weighted sagittal images covering the entire brain were obtained with a magnetization-prepared rapid gradient echo sequence: slices per slab: 192, slice thickness: 0.9 mm, gap: 0.45 mm, repetition time: 2530 ms, echo time: 2.4 ms, inversion time: 1100 ms, field of view: 256 mm × 256 mm, flip angle: 7°, and matrix: 256 × 256.

### Pre-processing of fMRI data

Pre-processing of fMRI data was performed using the Statistical Parametric Mapping software package (SPM8, http://www.fil.ion.ucl.ac.uk/spm) and the Data Processing Assistant for Resting-State fMRI Data Analysis Toolkit (DPARSF, http://resting-fmri.sourceforge.net). The first 10 volumes of each participant’s data set were discarded to allow for magnetization equilibrium and environment adaptation. The remaining 230 images were corrected for time delay between slices and for rigid-body head movement by co-registering them to the first image. None were excluded due to excessive motion (translation > 2.5 mm or rotation > 2.5 degrees). Also, there was no significant difference in the frame-wise [24] displacement between groups (t = 1.18, P = 0.25). Subsequently, the functional images underwent motion correction and were then spatially normalized to the Montreal Neurological Institute (MNI) space using a unified segmentation algorithm [25]. The data were re-sampled to an isotropic resolution of 3-mm using the parameters estimated during unified segmentation. Finally, the normalized images were spatially smoothed using an isotropic Gaussian filter with a full-width-half-maximum (FWHM) of 6 mm to reduce spatial noises.

### Independent component analysis

The ICA was performed using the free soft GIFT (http://icatb.sourceforge.net/, version 2.0h) for all fMRI data with three distinct stages [26]: First, a three-step principal component analysis (PCA) was used to decompose the data set into 34 components. Subsequently, the independent components (ICs) were estimated using the Informax algorithm [27]. Finally, the group ICA 3 (GICA3) approach, a reliable method to explore ICs [28], was used to back-reconstruct the individual subject components. After back-reconstruction, the time-courses of ICs and spatial ICs for each participant were acquired, and the subject-specific spatial maps were transformed to z scores. Each individual z map was entered into a random-effect two-tailed one-sample t-test to determine the group spatial map and the corresponding spatial pattern of each Resting-State Network (RSN) in SPM8 (P < 0.001, FDR corrected). Then, a two-tailed two-sample t-test was performed to compare the coactivation difference within each RSN between PSPD patients and HCs with a statistical significance level of P < 0.001 and cluster > 270 mm^3^ (uncorrected), with gender and age as nuisance covariates.

### Functional network connectivity analysis

The ICs are independent in spatial domain, however, the time-courses of ICs are not independent in time domain and exhibit significant temporal dependence among ICs [18]. Compared to the traditional FC analysis, which focuses on the correlation between a seed region and the remaining regions of the whole brain, the present study primarily focused on the temporal correlation between different RSNs. The present study by the visual inspection identified 12 meaningful RSNs (the detail descriptions see in the Result part) which have been reported in the previous studies [18,29–31]. The time-courses of 12 RSNs for each participant were extracted from the ICA analysis, and the Pearson’s correlation coefficient of the time-courses of each pair of the 12 RSNs, namely the functional network connectivity (FNC) [18], were calculated and normalized with Fisher r-to-z transformation. To compare the FNC differences between PSPD patients and HCs, a two-tail two-sample t test was performed with a statistical significance level of P < 0.05 (FDR corrected).

### Correlation analysis

To determine the relationship between FC with significant difference between groups and clinical characteristics, the Spearman correlation (nonparametric) analysis was performed between the FCs with significant difference and VAS, SAS, SDS and duration of illness in PSPD patients. A value of P < 0.05 was considered statistically significant.

## Results

### Resting-state networks

**Fig 1:**
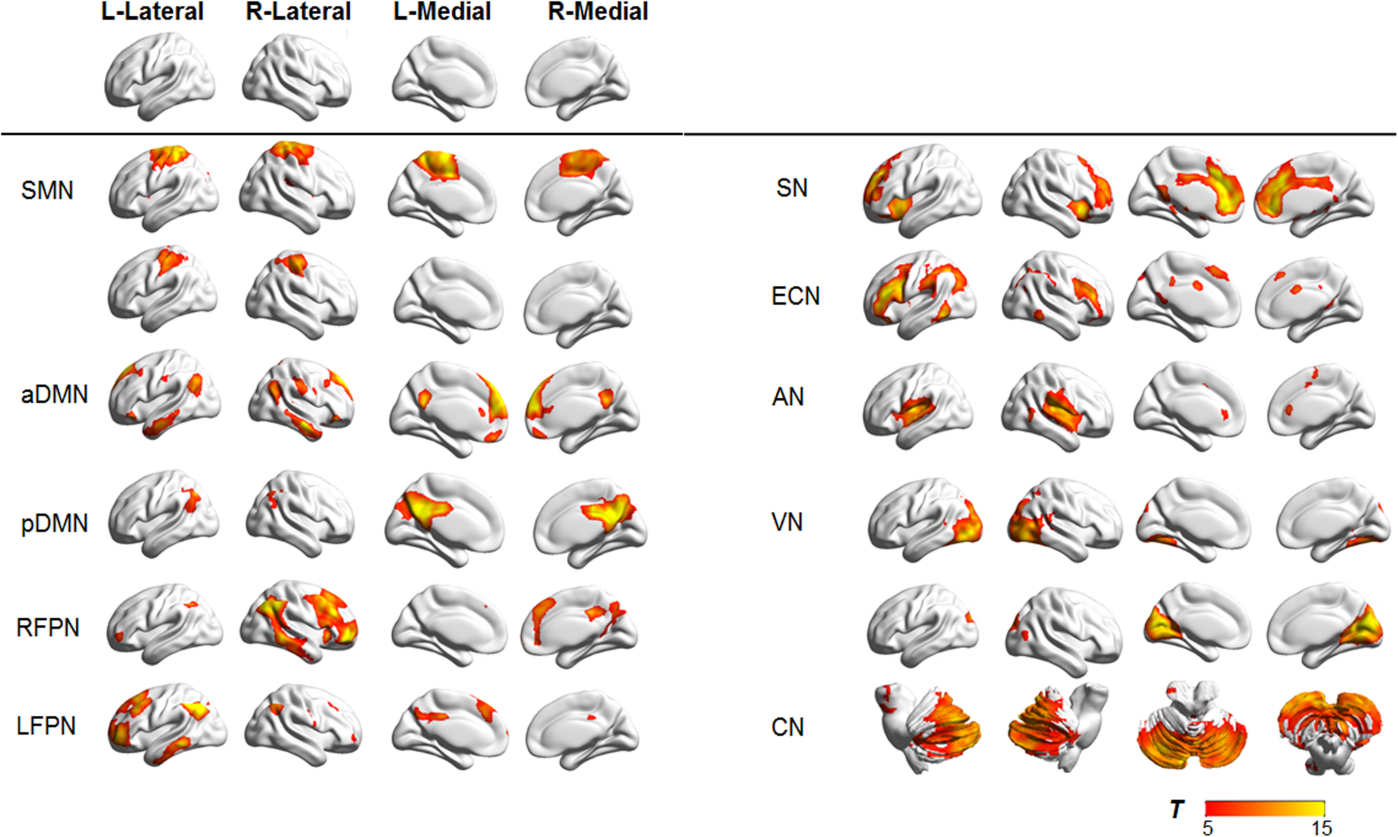
Cortical representation of brain networks identified in an independent component analysis. SMN: sensorimotor network; aDMN: anterior default-mode network; pDMN: posterior default-mode network; RFPN: Right frontoparietal network; LFPN: Left frontoparietal network; SN: Salience network; ECN: Executive control network; AN: Audio network; VN: Visual network; CN: Cerebellum network. L: left; R: right. Color bar represents the t values ranging from 5 to 15. Data are displayed on the lateral, medial surfaces of each hemisphere.

In 34 ICs estimated from ICA, the identified 12 ICs were consistent with RSNs described in previous studies [18,29–31](Fig 1). The sensorimotor network (SMN) includes the bilateral pre- and postcentral gyrus (Pre/PostCG), and supplementary motor areas (SMA). The anterior default-mode network (aDMN) consists of the mPFC and PCC/precuneus, and the posterior default-mode network (pDMN) consists of bilateral lateral parietal cortices (LPC) and precuneus. The frontoparietal network (FPN) is mainly involved in the dorsolateral prefrontal cortex (DLPFC) and posterior parietal cortex (PPC). The executive control network (ECN) includes the bilateral DLPFC, ventrolateral PFC (VLPFC), dorsomedial PFC, and LPC. The salience network (SN) is composed of the bilateral VLPFC, anterior insula (AI), and dorsal ACC (dACC). The auditory network (AN) primarily includes the bilateral superior temporal gyrus (STG). The visual network (VN) mostly includes the bilateral occipital lobe (OL). The cerebellum network (CN) includes the bilateral cerebellum (Cb).

### Altered coactivations of RSNs in PSPD patients

**Fig 2:**
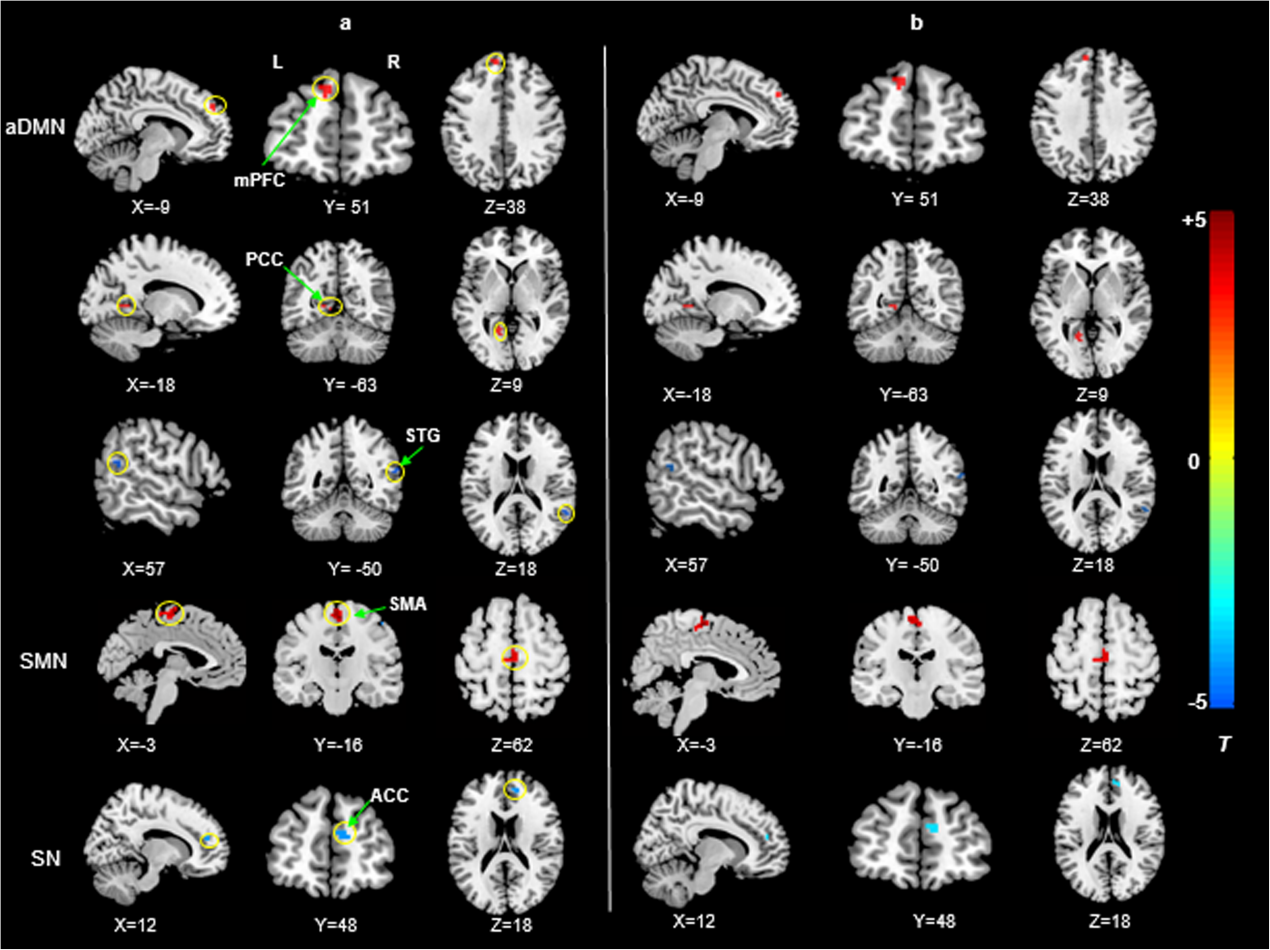
**Brain regions with significant differences between PSPD patients and HCs in the intra-network connectivity without (a) and with (b) removal of the effect of age and gender.** aDMN: anterior default-mode network; SMN: sensorimotor network; SN: Salience network. Red and blue denote higher and lower coactivations, respectively, in PSPD patients as compared to HCs, and the color bars indicate t-value (P < 0.001 and cluster size > 270 mm^3^, uncorrected).

Compared with HCs, PSPD patients showed decreased coactivations in the right STG within aDMN and the right ACC within SN while increased coactivations were found in the bilateral SMA within SMN, and the left mPFC and PCC within aDMN (Fig 2 and Table 2) (P < 0.001 and cluster > 270 mm^3^, uncorrected). These significant differences within networks between groups disappeared when the results were corrected by the FDR (P < 0.05).

**Table 2:** Regions showing significantly different coactivations within RSNs between groups.

Regions	RSN	BA	MNI coordinates (X,Y,Z)			Cluster (mm^3^)	t
PSPD < HC							
superior temporal gyrus	aDMN	40	57	-48	15	270	-4.09
anterior cingulate cortex	SN	9	12	48	18	297	-3.96
PSPD > HC							
supplementary motor area	SMN	6	3	-15	63	1809	4.97
posterior cingulate cortex	aDMN	31	-18	-63	9	351	3.82
medial prefrontal cortex	aDMN	9	-9	51	36	324	4.67

RSN represents the corresponding brain network of the regions. aDMN: anterior default-mode network; SMN: sensorimotor network; SN: Salience network.

### Altered FNCs in PSPD patients

**Fig 3:**
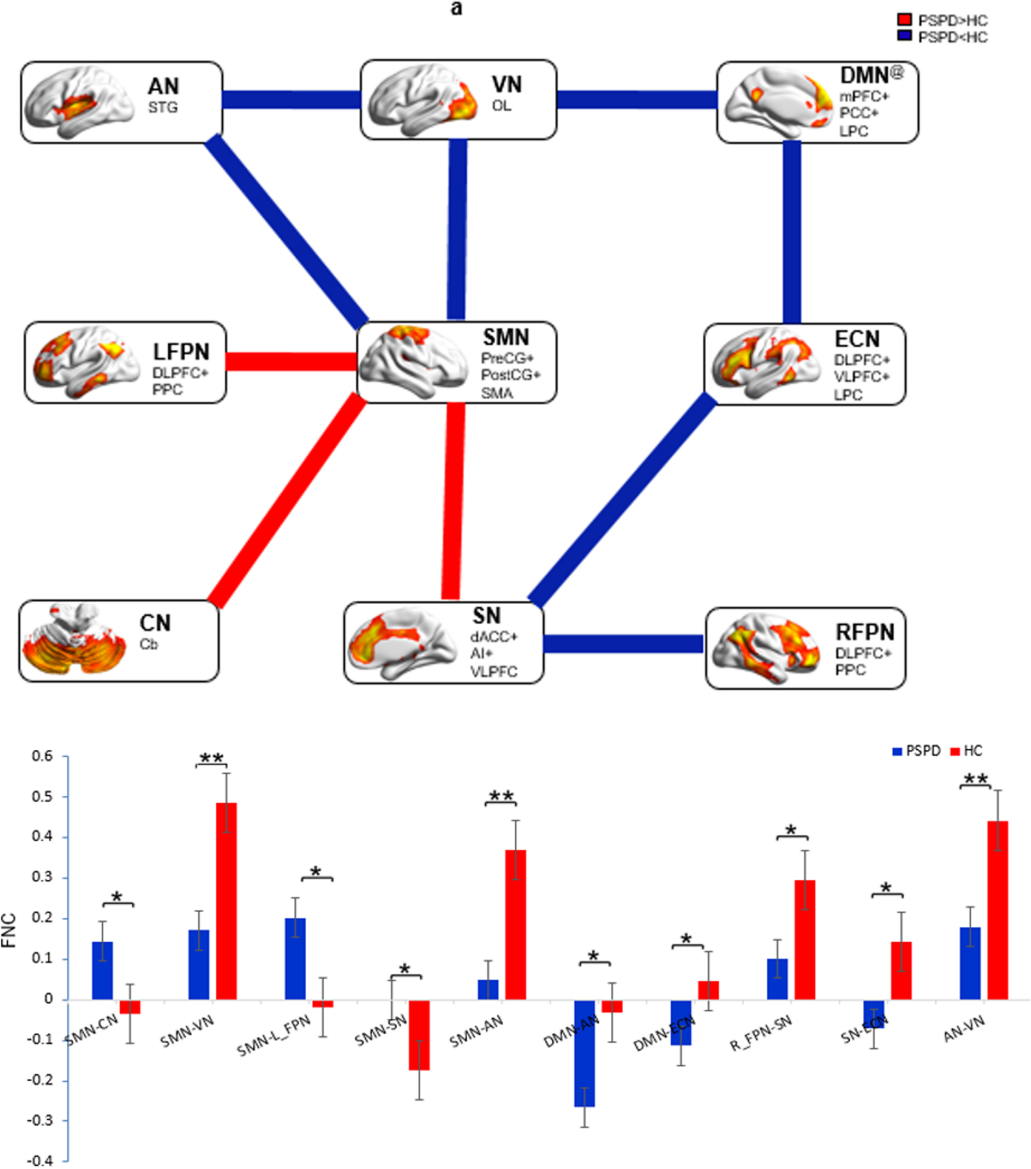
**Inter-network function changes between groups (a) and the quantitative display of differences (b).** SMN: sensorimotor network; DMN: Default-mode network; LFPN: Left frontoparietal network; RFPN: Right frontoparietal network; SN: Salience network; ECN: Executive control network; AN: Audio network; VN: Visual network; CN: Cerebellum network. @ represents that the DMN is the combination of aDMN and pDMN. The blue line between DMN and VN represents the connectivity between aDMN and VN, and the blue line between DMN and ECN represents the connectivity between pDMN and ECN. The y-coordinate in the subfigure b indicates z-score. Red and blue denote higher and lower FNC, respectively, in PSPD patients as compared to HCs, and the color bars indicate t-value (*indicate P < 0.05 and **indicate P < 0.01, FDR corrected).

As compared to HCs, PSPD patients showed significantly reduced FNCs in the SMN-VN and -AN, ECN-SN and -pDMN, AN-VN and -aDMN, RFPN-SN. Additionally, significantly enhanced internetwork connectivity was observed in the SMN-LFPN, and -SN and -CN (P < 0.05, FDR corrected). When the threshold was P < 0.01 (FDR corrected), only decreased FNCs between SMN and VN as well as AN were survived in PSPD patients (Fig 3).

### Correlation analysis between altered FC and clinical assessments

**Fig 4:**
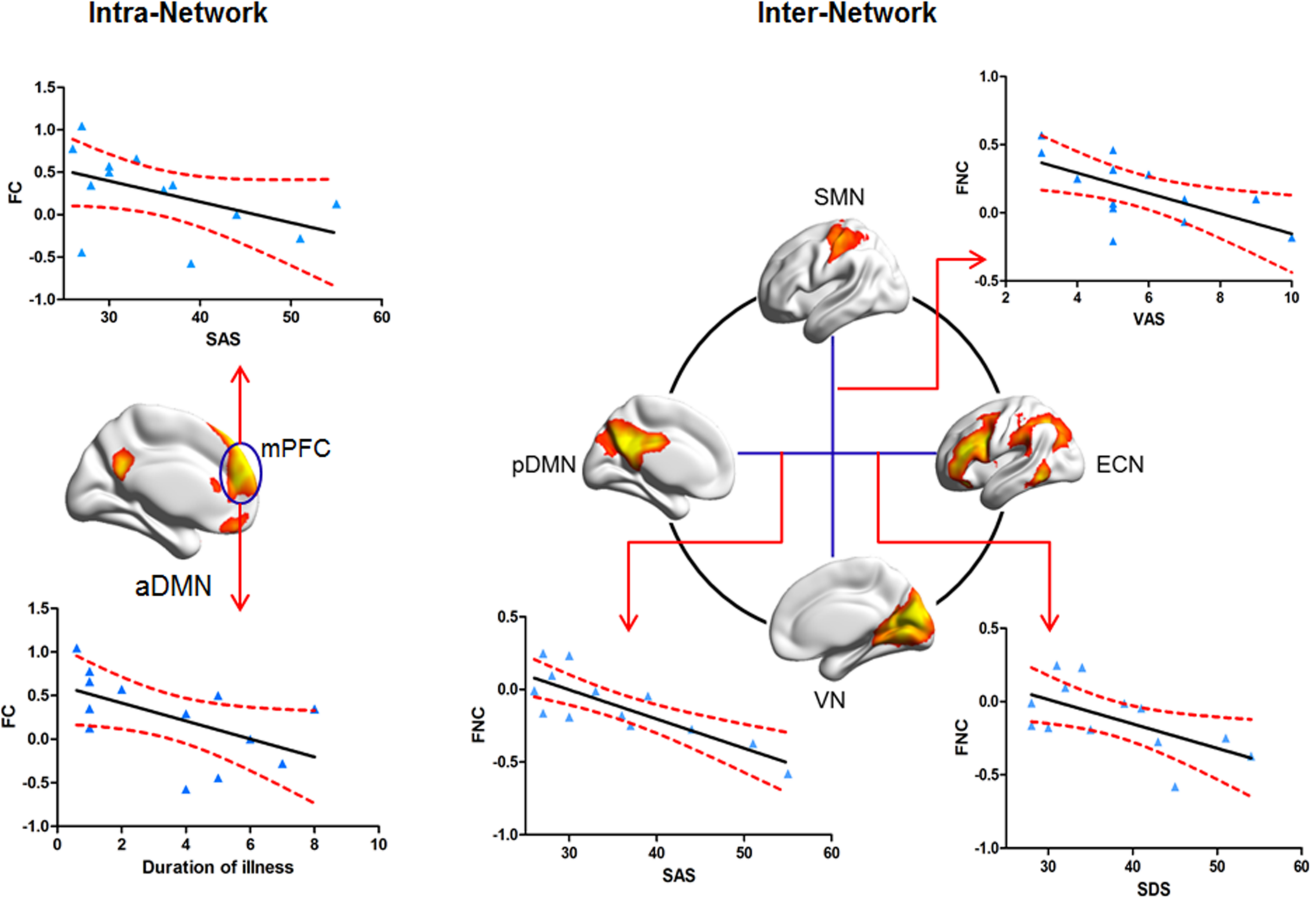
Significantly negative correlations between FC and clinical assessments. mPFC: medial prefrontal cortex; SMN: sensorimotor network; aDMN: anterior default-mode network; pDMN: posterior default-mode network; ECN: Executive control network; VN: Visual network (P < 0.05, uncorrected).

In the intra-network, significant negative correlations between mean coactivation of mPFC within aDMN and SAS (r = -0.57, P = 0.043) as well as duration of illness (r = -0.62, P = 0.025) were found in PSPD patients. For FNCs between networks, the mean FNC of pDMN-ECN in the PSPD patients was negatively correlated with SAS (r = -0.79, P = 0.001) and SDS (r = -0.66, P = 0.014), and the mean FNC of SMN-VN was negatively correlated with VAS (r = -0.56, P = 0.019) (uncorrected, Fig 4). However, there was no positive correlation.

### Reproducibility

**Fig 5:**
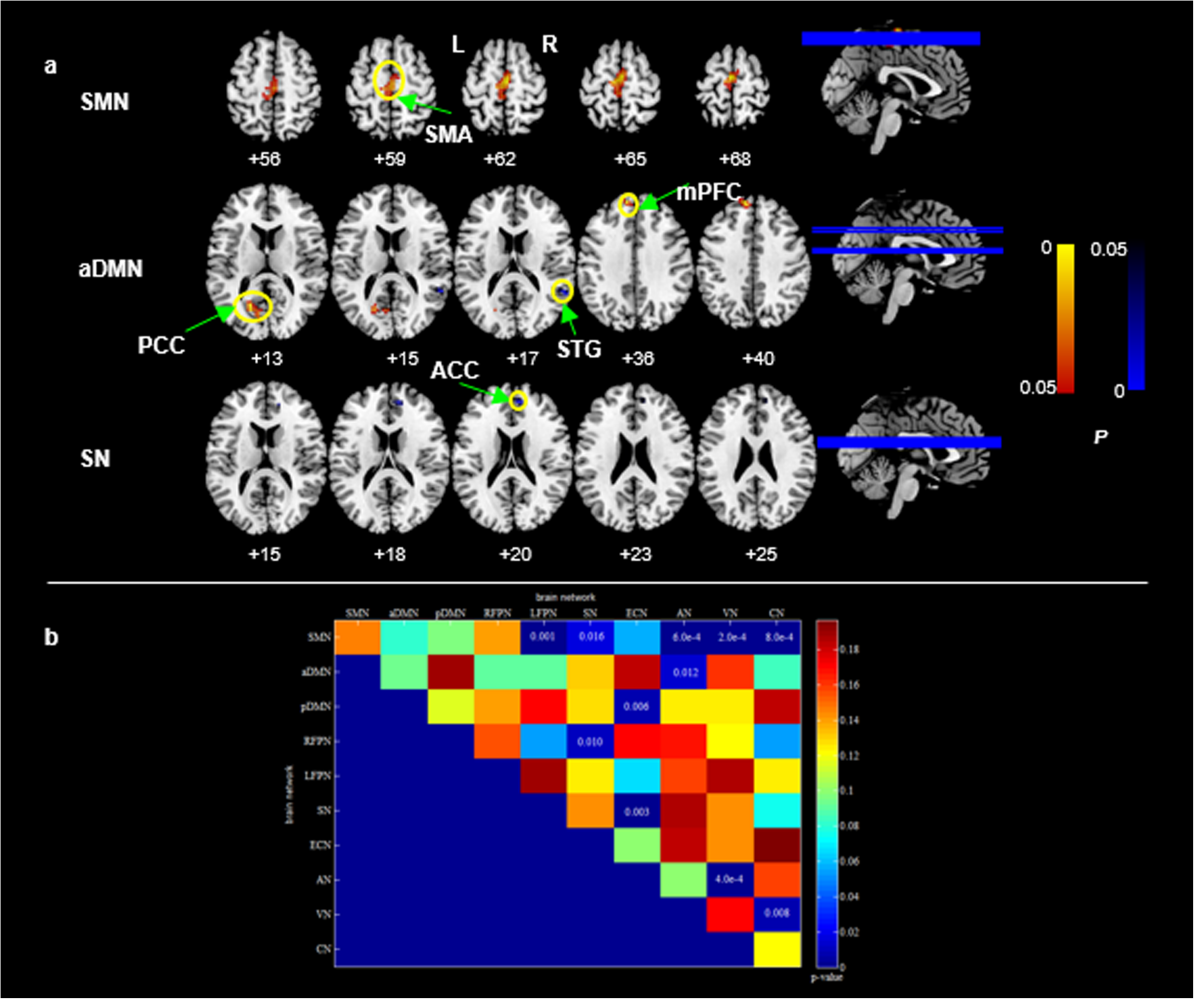
**Permutation test in the intra- (a) and inter-network (b) connectivity.** a: yellow circle represents the brain regions with between-group difference in intra-network. Hot and blue color show the higher and lower coactivations within network in the PSPD group compared with HCs, respectively. Color bar represents the p value (FWE corrected). b: the x and y coordinates represent the brain network. Color bar represents p value (uncorrected). L: left; R: right. aDMN: anterior default-mode network; SMN: sensorimotor network; SN: Salience network.

Due to the small sample size in the present study, the reliability of these findings on FC may be challenged. Therefore, to validate the reproducibility and robustness of all findings on FC in the current study, the group comparisons were performed again using a permutation-based non-parametric test (5, 000 permutations; covariates: age and gender). Multiple comparisons across space were corrected using the threshold-free clustering enhancement (TFCE) method [32]. The relatively high-reproducible patterns of FC changes for the intra-network (p<0.05, FWE corrected) and inter-network (p<0.05, uncorrected) across the permutation test as well as the original test reported were showed in Fig 5. The original results were completely replicated except for the decreased FNC of VN-aDMN in PSPD group compared with HCs.

## Discussion

As far as we know, this was the first study to detect the altered intra- and inter-network FCs in PSPD patients. Results in the present study support our hypothesis that PSPD patients exhibit the changes in FCs related to pain when compared to HCs. Significantly decreased coactivations were found in the right ACC within the SN and the right STG within the aDMN in PSPD patients, whereas increased coactivations were observed in the bilateral SMA within the SMN and both the left mPFC and PCC within the aDMN. The reduced FNCs were found in the SMN-VN and -AN, ECN-SN and -pDMN, AN-VN and -aDMN, and RFPN-SN, whereas the FNCs between SMN-LFPN and -SN and -CN were enhanced. Moreover, significant negative correlations were found between mean coactivation of mPFC within the aDMN as well as the FNC of the pDMN-ECN and the SAS, between the FNC of the SMN-VN and the VAS, between the FNC of the pDMN-ECN and the SDS, and between mean coactivation of mPFC within aDMN and duration of illness. These findings provide a novel insight for us to understand the large-scale functional reorganization in PSPD patients.

### Altered RSN coactivations in PSPD patients

Compared with HCs, PSPD patients showed increased coactivation in the bilateral SMA within the SMN. The SMA belongs to a part of sensorimotor cortex, which is mainly involved in sensorimotor integration, voluntary movements control, and motor-related information processing [5]. The structure base of pain processing includes multiple ascending pathways projecting to several brainstem and cortical regions, in some of which SMA is anatomically interconnected with S1 as well as ACC and associated with the precise coding of the intensity of nociceptive stimulation [33]. A recent review of structural brain imaging on chronic pain suggests that the gray matter is changed in the motor cortex (SMA or M1) of patients as compared to controls in a number of studies [34]. In the studies on somatoform pain disorders, more activations induced by pain are found in the SMA of patients compared with controls [35], and significant change in the signal intensity is detected in this region as responses to pain perception between patients and controls [36]. Therefore, we speculate that the increased coactivations of bilateral SMA within SMN in our study may be closely related to abnormal sensory-discriminative processing and the coding of pain intensity in PSPD patients. Moreover, it should be acknowledged that patients with a long course of pain and very high pain scores often reduce their exercise levels, which may be ascribed to their functional changes in SMN.

PSPD patients also showed increased coactivations in the left PCC and mPFC while decreased coactivation in the right STG within the DMN. The alterations of the DMN caused by chronic pain have been reported in many studies. For instance, a recent study by the resting-state fMRI demonstrated that the ReHo values of DMN regions in healthy subjects were increased after a painful stimulation [6]. The power spectra analysis showed that the BOLD fluctuations were higher in the DMN than in other brain regions of patients with chronic pain disorder, especially in the high-frequency band [6,37]. In a positron emission tomography (PET) study, increased regional cerebral blood flow (rCBF) of the bilateral PCC was found in patients with somatoform pain disorder [38]. A voxel-based morphometry (VBM) analysis also revealed decreases of gray matter density in the mPFC and PCC of pain patients compared to controls [39]. These findings consistently suggest that chronic pain commonly causes disruptions of DMN in the function and structure, which may underlie the cognitive and behavioral impairments accompanying chronic pain [40]. Corroborating these findings, the abnormal coactivations within DMN were noted in PSPD patients in the present study. More importantly, significantly negative correlations were found between mean coactivations of mPFC and SAS and duration of illness. This indicates that the changed coactivations are associated with the processing of pain involving emotionally intense information in PSPD patients.

ACC is an important brain area of the medial pain system, which primarily receives extensive projections from the mediodorsal thalamic nucleus and broadly connects with relevant regions of the descending modulation system [3]. Numerous studies have shown that aberrant signals, such as the reduced gray matter [34], ReHo [41], regional cortical activity [42] and increased rCBF [43], are commonly observed in the ACC of patients with chronic pain as compared to HCs. Studies on pain stimulation in healthy subjects have revealed that the painful heat induces a significant activation in the ACC using functional MRI and PET [44,45]. This activation is consistent with the encoding of perceived unpleasantness [45]. An fMRI study on heat stimulation suggests that the experimentally induced pain activation in the ACC correlates negatively with the intensity of clinical pain in patients [46], and the positive correlation is also found between blood flow of ACC and both pain sensation and discomfort in another PET study [47]. Using FC as an index, a recent functional study showed that the rostral ACC plays a crucial role in placebo analgesia, in which increased FC response to endogenous pain control [20]. These findings suggest that the ACC is related to the pain transmission and control of pain processing. Therefore, the decreased coactivations in the ACC within the SN in the present study may be associated with the dysfunctions of sensation and pain control in PSPD patients.

### Altered FNCs in PSPD patients

The present study also investigated how pain-related FNCs were changed in PSPD patients at rest. With the use of ICA approach, the SMN, bilateral FPN, ECN, DMN, SN, CN, VN, and AN were isolated. The SMN primarily involves in the sensory-discriminative aspect of pain processing [4,5]. Our results demonstrated that increased FNCs were observed between SMN and high-order networks (CN, SN and LPFN) while decreased FNCs were found between SMN and low-order networks (AN and VN). Most importantly, a significantly negative correlation was also found between FNC of SMN-VN and VAS. The strong interactions of SMN with the LFPN, SN, CN, VN and AN suggest that sensory-discriminative processing of pain may be highly related to the affective processing, cognitive control and weak visual as well as auditory perception. This may explain the influence of various subjective states for PSPD, such as anxiety, sadness, and individual predictions on the perception of pain via sensory systems. Hence, patients suffering from PSPD may be able to benefit from the psychological interventions that focus on the disturbed affect regulation and aim to enhance emotional awareness [9].

In PSPD patients, abnormal FNCs were also found among high-order networks, including ECN, DMN, SN and LFPN. These networks commonly involve the processing of emotion, attention, control and cognition in human brain [18,29,30]. Studies demonstrate that the FPN primarily mediates attention, working memory, and higher order cognitive processes [18] while the DMN is related to the cognitive control of emotions and self-referential processing, and memory encoding [48]. ECN acts as a feasible modulator between those two networks [49], which involves the cognitive control over both emotional and non-emotional materials [50]. Additionally, the SN, which identifies salient stimuli from the vast and continuous stream of sensory stimuli, has a critical and causal function in switching between ECN and DMN across task paradigms and stimulus modalities, and modulating information flow across other brain networks involved in attention processing and cognition [51]. In the present study, the uncoupled FNCs were found in the ECN-pDMN and -SN, SN-RFPN of PSPD patients, and significantly negative correlations were noted between the FNC of pDMN-ECN and SAS as well as SDS, which reflects that atypical FNCs of these networks in PSPD patients may be related to some pathophysiological mechanisms underlying the functionally impaired emotional state decoding and cognitive control in PSPD patients.

A recent resting-state fMRI study on somatoform pain disorder shows significant FNCs between the cingular-insular network and SMN, the aDMN and pDMN/SMN, and the pDMN and SMN in both patient and control groups, respectively, while no significant differences were found in FNCs between the two groups [20]. In contrast to this study, the present study showed that PSPD led to significant changes in FNCs among pain-associated networks at rest in patients as compared to HCs. Because chronic pain has been shown to be a strong disruptor of intra-network FC within the sensory, affective and cognitive neural systems, these FNC findings suggest that functional networks of the brain may be not operated independently in encoding different aspects of pain but are highly interactive. Moreover, when the multiple correction is more restricted (P < 0.01), only the differences in FNCs among low-order perception networks (SMN, VN and AN) persisted between groups. We speculate that these effects of PSPD on the large-scale functional networks may mirror the underlying neural mechanism that sensory attributes of pain can be conceived as important causal links in the production of pain-related emotional [33] and cognitive disturbances modulated by some high-order networks (ECN, DMN, SN and FPN). These functional reorganizations in the network scale may be helpful for interpreting the complex experience of pain involving sensory-discriminative, affective-motivational and cognitive-evaluative components in PSPD patients [52].

It is worth to note that our recent study using the ReHo method found, compared with the HC group, PSPD patients exhibited decreased ReHo in the bilateral primary somatosensory cortex, posterior cerebellum, and occipital lobe, while they displayed increased ReHo in the prefrontal cortex (PFC) and default mode network. Additionally, significant positive correlations were found between the mean ReHo of both right IPL and left supramarginal gyrus and participants’ SAS scores, and between the mean ReHo of the left middle frontal gyrus and VAS scores. Consistent with this study, the current study also found those changed coactivations within DMN and SMA of primary somatosensory cortex within SMN in PSPD patients. Additionally, we found the altered coactivation in the ACC within SN and numerous disruptions in inter-network FCs negatively correlated with the clinical assessments in PSPD patients among high- and low-order networks. Although both the current study and the published work [15] belong to the same project and the resting-state fMRI data were identical in two studies, their purpose and method of research were significantly different. The study [15] used the Regional Homogeneity method to assess the difference in regional spontaneous activity between PSPD patients and HCs at rest, whereas the present study utilized the independent component analysis to explore the intra- and inter-network functional connectivity changes in the PSPD patients compared with HCs. Therefore, these two studies revealed the PSPD pathologies from different perspective.

Although we utilized the ICA method to reveal abnormal functional connectivity in the intra- and inter-networks associated with pain in PSPD patients, the present study still had several issues. First, the sample size is relatively small and participants’ self-reported pain was diffuse due to the characteristics of PSPD. This may limit the robustness of the findings. Therefore, a larger sample size is needed to confirm our findings in the future. Second, some treatments (e.g., antidepressants) which influenced the pain perception of PSPD may affect our results, and this was not included as an exclusion criterion. Finally, the results within RSNs were not survived after the multiple comparisons correction. Therefore, we adopted a compromised threshold (P < 0.001, uncorrected) to display the trends of the group difference.

## Conclusion

The present study investigated the alterations of RSN connectivity in PSPD patients, and found significantly altered coactivations within the SMN, aDMN and SN, and FNCs between the SMN and LFPN, SN, CN, VN as well as AN, and between the ECN and SN as well as pDMN, between the RFPN and SN, between the AN and VN as well as aDMN between PSPD patients and HCs. The large-scale functional reorganization in PSPD patients may be helpful for us to understand the pathological mechanism underlying the dysfunctions of PSPD in multi-dimension aspects of pain.

## Supporting information

S1 Table Demographic characteristics and clinical assessment of individual for PSPD patients and HCs. (DOCX)
