# Supplementary material for: Altered resting-state intra- and inter- network functional connectivity in patients with persistent somatoform pain disorder
Source: PLoS One. 2017 Apr 28;12(4):e0176494. doi: 10.1371/journal.pone.0176494 (PMC5409184; doi:10.1371/journal.pone.0176494)
Supplement: S1 Table — (DOCX) [file pone.0176494.s001.docx]

**Table 1** Demographic characteristics and clinical assessment of individual for PSPD patients and HCs

| **ID** | **Age (yr)** | **Gender** | **Handedness** | | **Duration (yr)** | **VAS** | **SAS** | **SDS** |
| --- | --- | --- | --- | --- | --- | --- | --- | --- |
| *PSPD patients* | | | | | | | | |
| 01 | 60 | F | R | 8 | | 3 | 28 | 32 |
| 02 | 43 | F | R | 1 | | 6 | 33 | 39 |
| 03 | 65 | F | R | 2 | | 4 | 30 | 35 |
| 04 | 63 | F | R | 0.6 | | 10 | 27 | 31 |
| 05 | 27 | M | R | 7 | | 7 | 51 | 54 |
| 06 | 43 | F | R | 5 | | 5 | 27 | 28 |
| 07 | 30 | M | R | 5 | | 5 | 30 | 34 |
| 08 | 33 | M | R | 1 | | 5 | 55 | 45 |
| 09 | 27 | M | R | 6 | | 9 | 44 | 43 |
| 10 | 48 | F | R | 1 | | 3 | 26 | 28 |
| 11 | 39 | F | R | 1 | | 5 | 37 | 51 |
| 12 | 62 | M | R | 4 | | 7 | 36 | 30 |
| 13 | 58 | F | R | 4 | | 5 | 39 | 51 |
| *HCs* | | | | | | | | |
| 01 | 43 | F | R | 0 | | - | - | - |
| 02 | 51 | F | R | 0 | | - | - | - |
| 03 | 33 | F | R | 0 | | - | - | - |
| 04 | 27 | M | R | 0 | | - | - | - |
| 05 | 26 | M | R | 0 | | - | - | - |
| 06 | 27 | M | R | 0 | | - | - | - |
| 07 | 27 | M | R | 0 | | - | - | - |
| 08 | 49 | F | R | 0 | | - | - | - |
| 09 | 42 | F | R | 0 | | - | - | - |
| 10 | 26 | F | R | 0 | | - | - | - |
| 11 | 53 | M | R | 0 | | - | - | - |
| 12 | 59 | M | R | 0 | | - | - | - |
| 13 | 55 | M | R | 0 | | - | - | - |
| 14 | 59 | M | R | 0 | | - | - | - |
| 15 | 55 | M | R | 0 | | - | - | - |
| 16 | 51 | M | R | 0 | | - | - | - |
| 17 | 48 | M | R | 0 | | - | - | - |
| 18 | 51 | M | R | 0 | | - | - | - |
| 19 | 38 | F | R | 0 | | - | - | - |
| 20 | 59 | F | R | 0 | | - | - | - |
| 21 | 64 | F | R | 0 | | - | - | - |
| 22 | 56 | F | R | 0 | | - | - | - |
| 23 | 60 | F | R | 0 | | - | - | - |
